# Different Associations Between the *IREB2* Variants and Chronic Obstructive Pulmonary Disease Susceptibility

**DOI:** 10.3389/fgene.2020.598053

**Published:** 2020-11-16

**Authors:** Qiaoli Zeng, Qikang Chen, Dehua Zou, Runmin Guo, Dawei Xiao, Shaohu Jiang, Riling Chen, Yajun Wang, Guoda Ma

**Affiliations:** ^1^Maternal and Child Research Institute, Shunde Women and Children's Hospital, Guangdong Medical University, Foshan, China; ^2^Department of Medicine, Shunde Women and Children's Hospital, Guangdong Medical University, Foshan, China; ^3^Department of Pediatrics, Shunde Women and Children's Hospital, Guangdong Medical University, Foshan, China

**Keywords:** chronic obstructive pulmonary disease, *IREB2*, polymorphism, meta analysis, susceptibility

## Abstract

**Background:** Iron responsive element binding protein 2 (*IREB2*) variants may be involved in the pathogenesis of chronic obstructive pulmonary disease (COPD). Recently, many studies have been performed on *IREB2* susceptibility variants, including rs2568494, rs2656069, rs10851906, rs12593229, and rs13180, associated with COPD. However, inconsistent findings have been reported. The aim of our research was to determine the association of *IREB2* SNPs with COPD.

**Methods:** A comprehensive meta-analysis was performed to accurately estimate the association between *IREB2* variants and COPD among four different genetic models.

**Results:** This meta-analysis included a total of 4,096 patients and 5,870 controls. Here, we investigated the 5 *IREB2* variants to identify COPD risk. Our results indicate that rs2568494 was associated with an increased risk of COPD for the dominant model (AA+GA vs. GG: OR = 1.150, 95% CI: 1.5–1.304, *P* = 0.029); rs2656069 was associated with a decreased risk of COPD for the recessive model (GG vs. AA+AG: OR = 0.589, 95% CI: 0.440–0.789; *P* = 0.000), additive model (GG vs. AA: OR =0.641, 95% CI: 0.441–0.931; *P* = 0.020), and allele model (G vs. A: OR = 0.812, 95% CI: 0.668–0.988; *P* = 0.037); and rs10851906 was associated with a decreased risk of COPD for the recessive model (GG vs. AA+AG: OR = 0.732, 95% CI: 0.560–0.958; *P* = 0.023) and additive model (GG vs. AA: OR = 0.777, 95% CI: 0.637–0.947; *P* = 0.012).

**Conclusion:** Our findings suggest that the *IREB2* rs2568494 minor alleles A may be a genetic factor in susceptibility to COPD. In addition, the minor alleles G of rs2656069 and rs10851906 appear to have a protective effect.

## Introduction

Chronic obstructive pulmonary disease (COPD) is a complex human genetic disease characterized by pulmonary dysfunction and incomplete reversibility of airflow obstruction (Rabe et al., 2007). It is predicted to become the fourth leading cause of death by 2030 (Mathers and Loncar, 2006). It is well-known that COPD is the result of interactions between genetic susceptibility and environmental factors. Although smoking is an important environmental factor in COPD, the COPD susceptibility of smokers varies, and the disease can also appear in non-smokers. These results suggest that genetic factors may be among the reasons for individual susceptibility.

The *IREB2* gene codes for an iron-binding protein (IRP2), which participates in maintaining iron homeostasis in human cells. A previous study reported that iron in the lungs has been shown to increase with age and that smokers had higher concentrations of iron in the lungs (Ghio et al., 1997). Thus, abnormalities in *IREB2* expression or functioning might contribute to iron metabolism disorders. This iron imbalance may lead to oxidative damage. Furthermore, an excess of iron has an influence on local inflammation in the lungs, which may be related to the pathogenesis of COPD. Interestingly, several genome-wide association studies have identified a *IREB2* genomic region associated with COPD (DeMeo et al., 2009; Hancock et al., 2010; Pillai et al., 2010). Moreover, the study reported that three *IREB2* SNPs were associated with COPD patients, and increased expression levels of *IREB2* mRNA have been detected in the lungs of smokers and COPD patients (DeMeo et al., 2009), which showed *IREB2* as a COPD susceptibility gene.

Over the past few years, many SNPs (rs2568494, rs2656069, rs10851906, rs12593229, and rs13180) in *IREB2* gene studies have different degrees of reproducibility. Numerous studies have investigated the relation to *IREB2* SNPs and COPD susceptibility (Chappell et al., 2011; Guo et al., 2011; Cho et al., 2012; Hardin et al., 2012; Zhou et al., 2012; Arja et al., 2014; Ding et al., 2015; Ziolkowska-Suchanek et al., 2015; Korytina et al., 2016; Nedeljkovic et al., 2018). However, the findings in these studies were inconsistent. Conflicting results may be due to the different populations and small sample sizes of each individual study. Therefore, the purpose of this meta-analysis was to collect all eligible studies to determine the association between *IREB2* gene polymorphism and COPD susceptibility.

## Materials and Methods

### Literature Search

The PubMed and Web of Science databases were systematically searched for relevant studies published before July 30, 2020, using the following key words: (1) “*IREB2*” or “rs2568494” or “polymorphism” and “COPD”; (2) “*IREB2*” or “rs2656069” or “polymorphism” and “COPD”; (3) “*IREB2*” or “rs10851906” or “polymorphism” and “COPD”; (4) “*IREB2*” or “rs12593229” or “polymorphism” and “COPD”; and (5) “*IREB2*” or “rs13180” or “polymorphism” and “COPD.” The search had no language restrictions. All studies were assessed by reading the title and abstract, and irrelevant studies were excluded. Then, the full texts of the remaining studies were assessed to determine their eligibility.

### Inclusion and Exclusion Criteria

The study inclusion criteria were as follows: (1) The study was a cohort or a case-control study; (2) The studies assessed the association between *IREB2* SNPs (rs2568494, rs2656069, rs10851906, rs12593229, and rs13180) and COPD; (3) COPD was diagnosed depending on the distinct clinical criteria. The inclusion criteria for stable COPD comprised airflow limitation as indicated by post-bronchodilator FEV1/FVC <70% and FEV1 ≤ 70% of normal predicted values. The severity of airflow limitation was classified according to the GOLD standard (Global Initiative for Chronic Obstructive Lung Disease) as follows: II-moderate, III-severe, and IV-very severe; (4) The control subjects were in Hardy-Weinberg equilibrium (HWE); (5) The selected studies provided SNP genotype amounts or sufficient data for calculation; and (6) The selected studies provided an odds ratio (OR) with a 95% confidence interval (CI) or sufficient data for calculation (He et al., 2015).

The exclusion criteria were as follows: (1) The study was not a cohort or a case-control study; (2) the study lacked particular raw or calculated data for OR with a 95% CI; (3) study on the repeatability (if the study population overlaps, only the study with the largest sample size was selected); and (4) the control subjects were not in a HWE.

### Data Extraction

The following data were independently extracted from the included studies and entered into a database to ensure the veracity of the data: first author, country, year of publication, origin, the numbers of COPD cases and controls, subjects' age and gender, BMI, pack years, FEV1 (%), FEV1/FVC, the distribution of genotype and alleles, ORs with 95% CI, or ability to calculate the OR and 95% CI.

### Statistical Analysis

HWE was examined by Pearson's chi-squared test (Shen et al., 2015; Zhang et al., 2015). Four genetic models were used in the study: the dominant model, the recessive model, the additive model and the allele model. Genetic heterogeneity was evaluated using the *Q-*test and *I*^2^-test. *I*^2^ statistics range from 0 to 100%. Significant heterogeneity was defined as *P* < 0.01 and *I*^2^ > 50% (Li et al., 2016; Liu et al., 2017; Han et al., 2019). ORs with corresponding 95% CIs were calculated using the fixed effects model (Mantel-Haenszel) when no significant heterogeneity was observed; otherwise, a random effects model was used. The *Z-*test was used to test the significance of the ORs. Additionally, Egger's and Begg's tests were used to assess publication bias. All statistical analyses were performed using STATA v.16.0 software (Stata Corporation, Texas, USA).

## Results

### Study Inclusion and Characteristics

**Figure 1:**
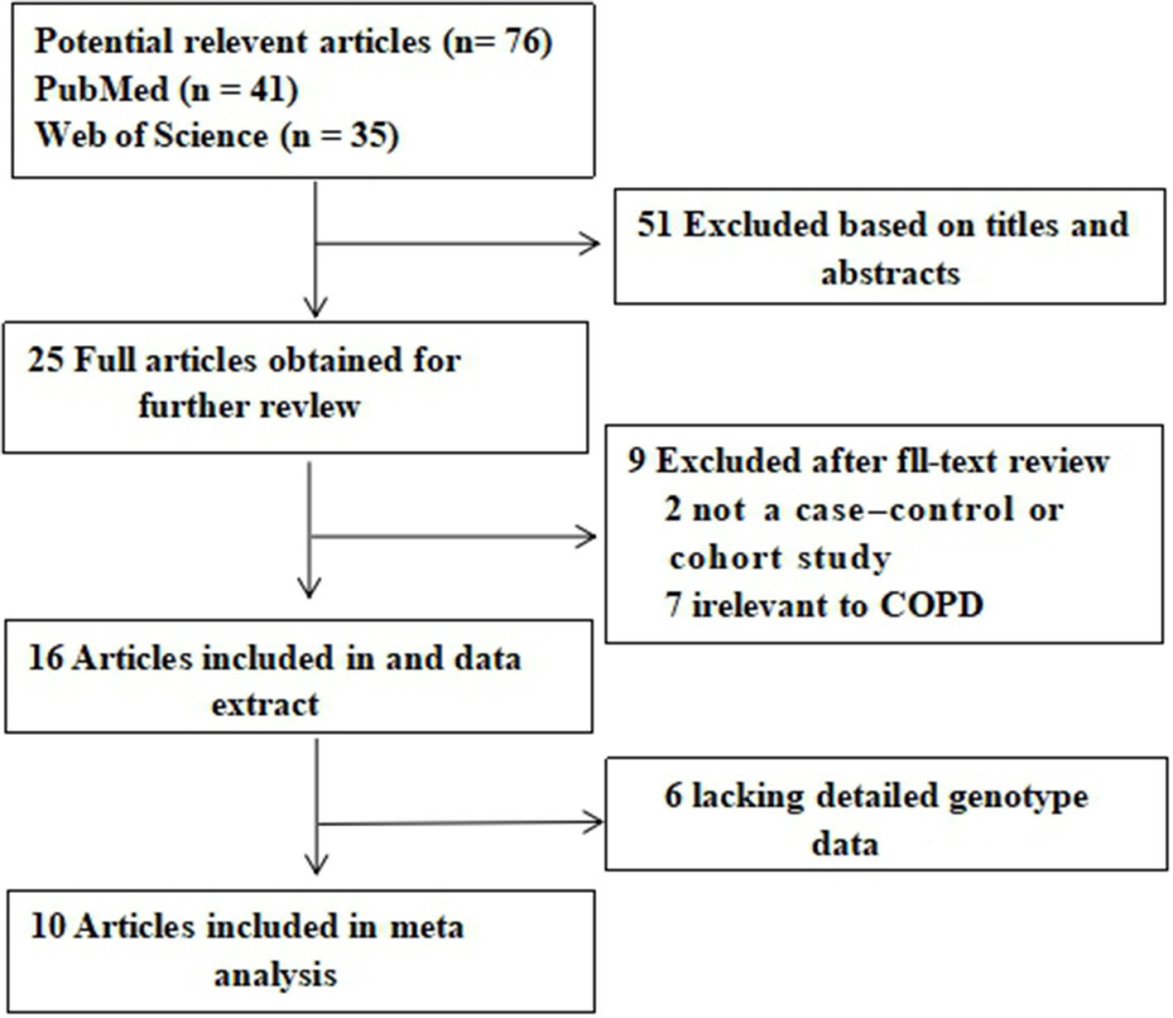
Flow diagram of the literature search and selection.

A total of 41 and 35 potential studies were retrieved from the PubMed and Web of Science databases. Using the inclusion and exclusion criteria, we selected 10 articles for meta-analysis. A flow chart of the study selection process is shown in Figure 1. Table 1 shows the major characteristics of each included study. Supplementary Tables 1–5 of the **Supplementary Materials** show the OR and 95% CI values of the four genotypes of each SNP. The control subjects of each study were in accordance with HWE.

**Table 1:** Characteristics of the studies included in the meta-analysis.

**Study Year**	**Origin**	**Study design**	**Sample size**		**HWE**	**Age**		**Gender**		**BMI kg/m**^****2****^		**Pack years**		**FEV1 (%)**		**FEV1/FVC (%)**	
			**Cases**	**Controls**	**Controls**	**Cases**	**Controls**	**Cases**	**Controls**	**Cases**	**Controls**	**Cases**	**Controls**	**Cases**	**Controls**	**Cases**	**Controls**
Guo et al. (2011)	Chinese	Case-control	275	434	yes	60.9	61.2	192/83	307/127	–	–	32.8	30.8	52.8	91.5	48.2	90.3
Chappell et al. (2011)	Caucasian	Cohort	1,002	900	yes	65.9	60.8	700/302	575/325	–	–	48.8	38.6	43.1	95.3	47.5	77.8
Zhou et al. (2012)	Chinese	Case-control	488	687	yes	62.7	60.9	–	–	22.5	22.4	33.8	29.2	64.2	102.4	53.6	79.3
Hardin et al. (2012)	Poland	Case-control	315	330	yes	61.95	58.28	220/95	222/108	26.7	27.72	44.5	33.37	30.4	102.5	36	77
Cho et al. (2012)	Norway	Cohort	863	808	yes	65.53	55.62	526/337	412/396	–	–	32	19.66	50.6	94.91	–	–
Arja et al. (2014)	Indian	Case-control	236	146	yes	63.2	61.1	236/0	146/0	19.8	22.01	43	48.2	36.8	71.7	55.4	86
Ding et al. (2015)	China	Case-control	200	401	yes	71.7	48.6	142/58	286/115	20	21.06	–	–			62	80
Ziolkowska-Suchanek et al. (2015)	Poland	Case-control	149	524	yes	–	–	107/42	379/145	–	–	–	–	–	–	–	–
Korytina et al. (2016)	Russia	Case-control	511	508	yes	62.74	58.82	452/59	457/51	25.8	27.06	44.6	38.54	41.7	130	58.66	87.94
Nedeljkovic et al. (2018)	Netherlands	Cohort	46	541	yes	61.9	59.3	–	233/308	–	–	34.3	19.9	–	–	63	78
Nedeljkovic et al. (2018)	Netherlands	Cohort	11	591	yes	68.2	67.6	–	249/342	–	–	33.7	19.6	–	–	63	78

*HWE, Hardy-Weinberg equilibrium; BMI, body mass index; FEV1, forced expiratory volume in 1s; FVC, forced vital capacity; (-), not applicable*.

### Heterogeneity Analysis

Genetic heterogeneity was evaluated using Cochran's Q statistic and *I*^2^ statistic.

#### rs2568494

**Figure 2:**
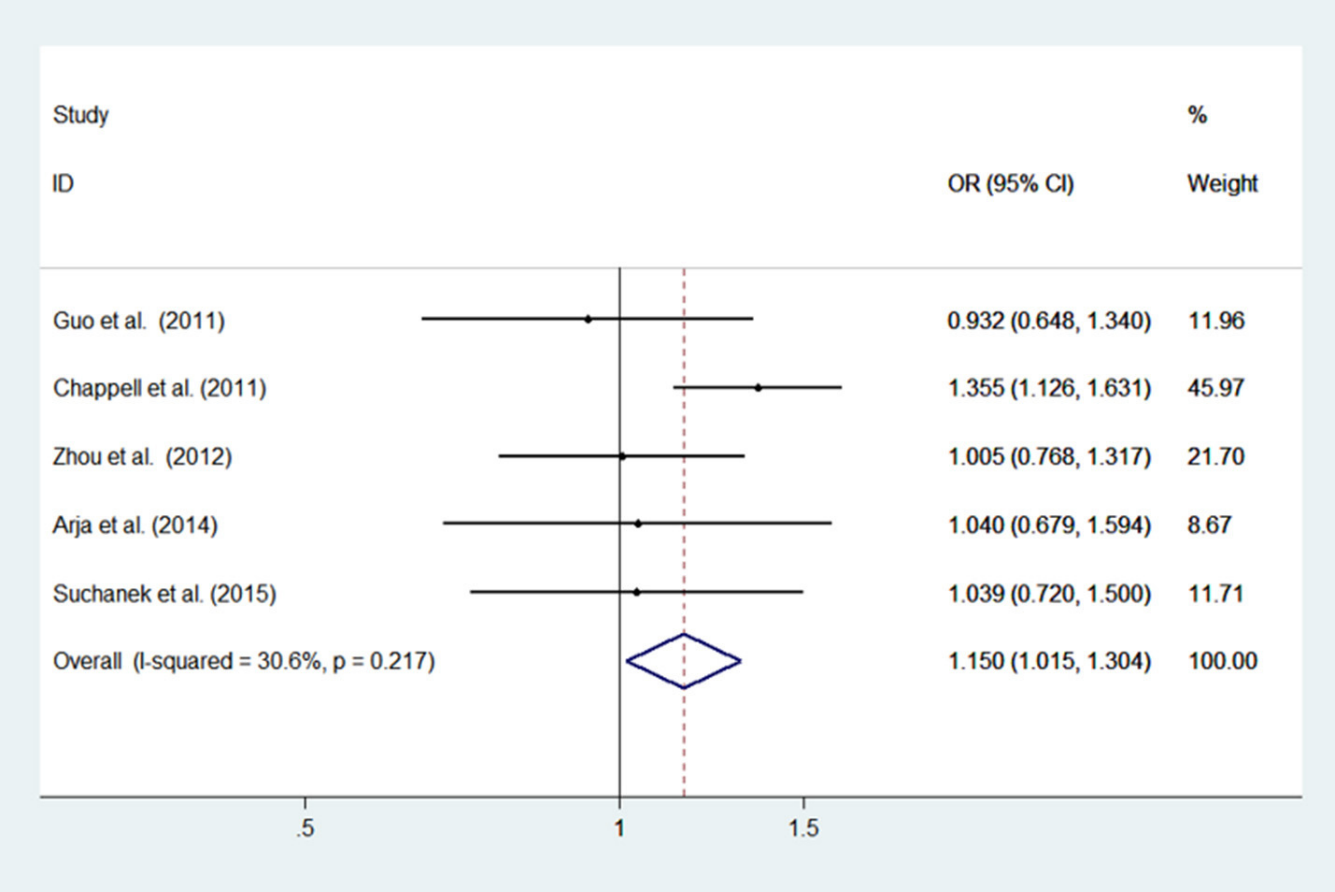
Meta-analysis with a fixed effects model for the association between the *IREB2* rs2568494 polymorphism and COPD susceptibility (dominant model, AA+GA vs. GG). OR, odds ratio; CI, confidence interval; I-squared, measure to quantify the degree of heterogeneity in meta-analyses.

Low heterogeneity among studies (Chappell et al., 2011; Guo et al., 2011; Zhou et al., 2012; Arja et al., 2014; Ziolkowska-Suchanek et al., 2015) was detected in the dominant model (AA+GA vs. GG: *I*^2^ = 30.6%, *P* = 0.217), and a moderate degree of heterogeneity was identified among studies under the recessive model (AA vs. GG+GA: *I*^2^ = 67.6%, *P* = 0.015), additive model (AA vs. GG; *I*^2^ = 66.2%, *P* = 0.019), and allele model (A vs. G: *I*^2^ = 60.3%, *P* = 0.039) (Figure 2, Supplementary Figure 1).

#### rs2656069

**Figure 3:**
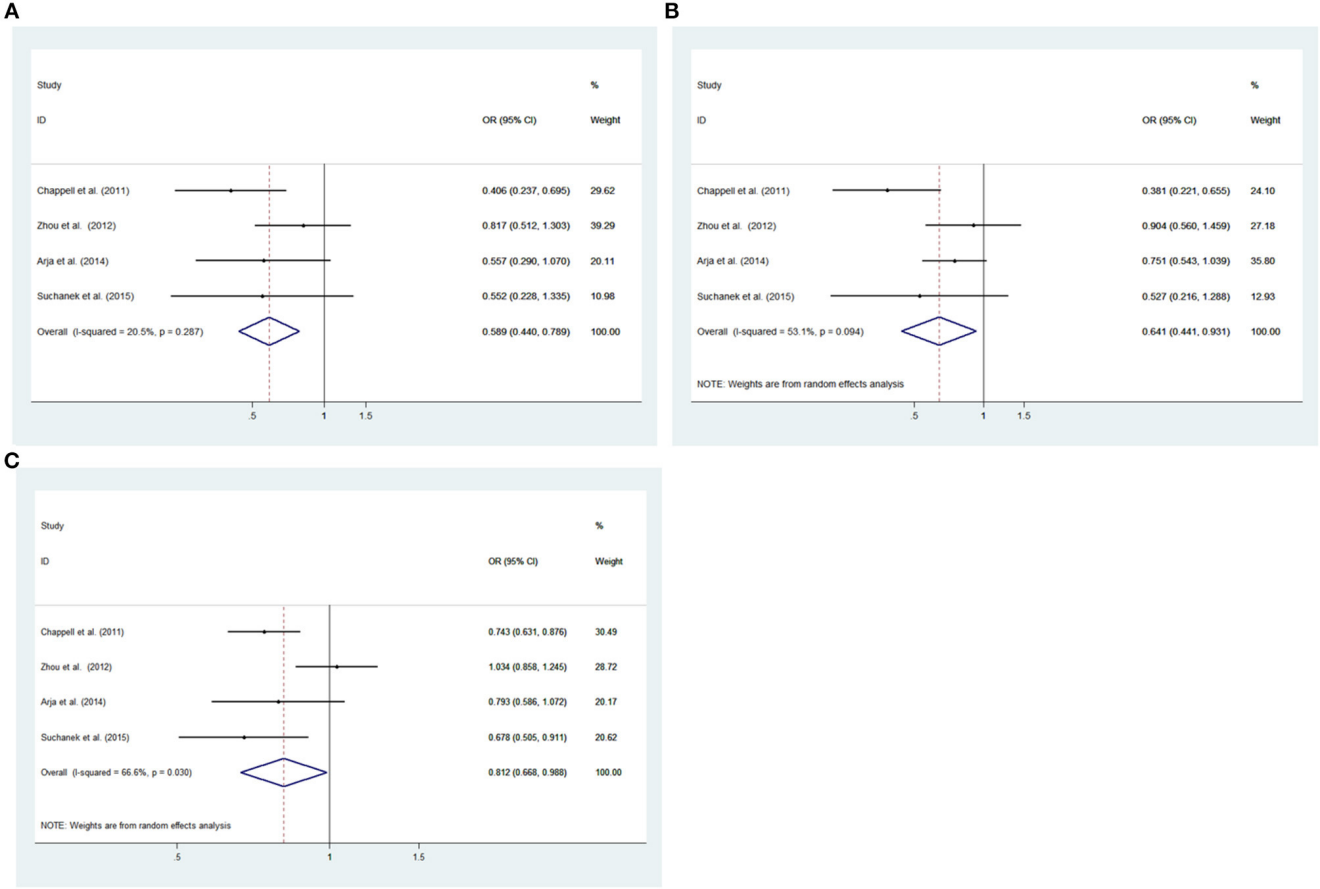
Meta-analysis for the association between the *IREB2* rs2656069 polymorphism and COPD susceptibility. **(A)** Recessive model, GG vs. AA+AG (fixed effects model). **(B)** Additive model, GG vs. AA (random effects model). **(C)** Allele model, G vs. A (random effects model). OR, odds ratio; CI, confidence interval; I-squared, measure to quantify the degree of heterogeneity in meta-analyses.

Low heterogeneity among studies (Chappell et al., 2011; Zhou et al., 2012; Arja et al., 2014; Ziolkowska-Suchanek et al., 2015) was detected in the recessive model (GG vs. AA+AG: *I*^2^ = 20.5%, *P* = 0.287), and a moderate degree of heterogeneity was found among studies under the dominant model (GG+AG vs. AA: *I*^2^ = 69.2%, *P* = 0.021), additive model (GG vs. AA; *I*^2^ = 53.1%, *P* = 0.094), and allele model (G vs. A: *I*^2^ = 66.6%, *P* = 0.030) (Figure 3, Supplementary Figure 2).

#### rs10851906

**Figure 4:**
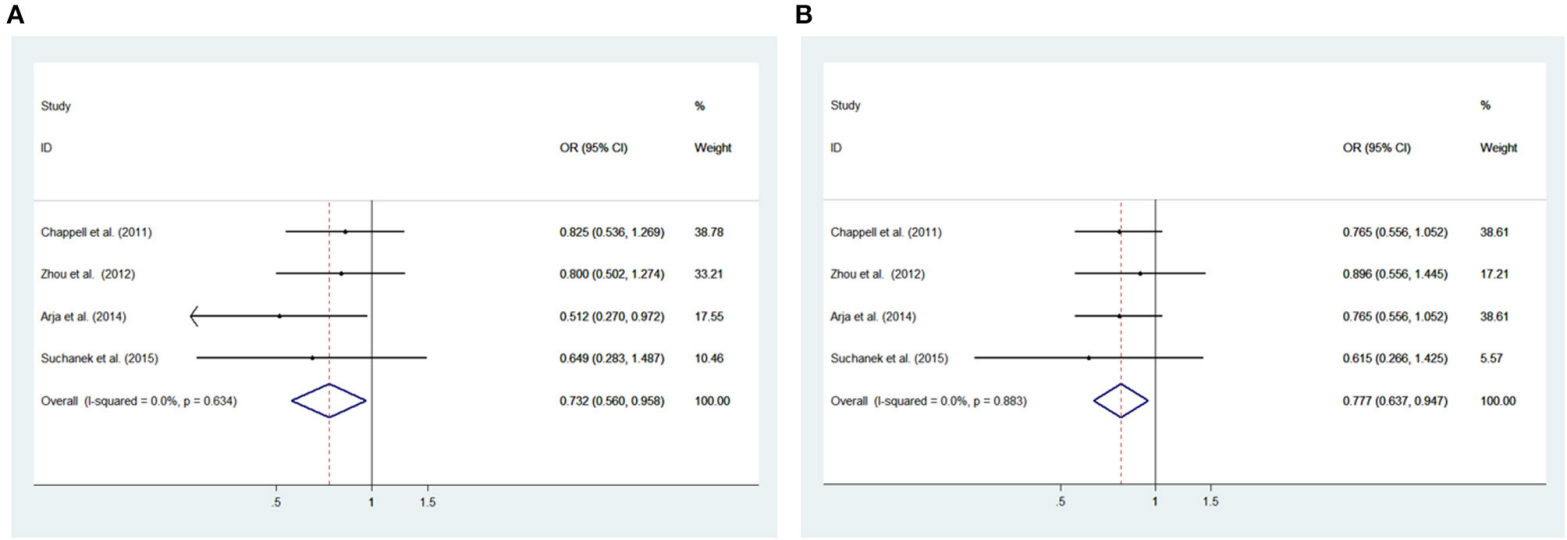
Meta-analysis with a fixed effects model for the association between the *IREB2* rs10851906 polymorphism and COPD susceptibility. **(A)** Recessive model, GG vs. AA + AG. **(B)** Additive model, GG vs. AA. OR, odds ratio; CI, confidence interval; I-squared: measure to quantify the degree of heterogeneity in meta-analyses.

Low heterogeneity among studies (Chappell et al., 2011; Zhou et al., 2012; Arja et al., 2014; Ziolkowska-Suchanek et al., 2015) was detected in the recessive model (GG vs. AA+AG: *I*^2^= 0.0%, *P* = 0.634), additive model (GG vs. AA; *I*^2^ = 0.0%, *P* = 0.883) and allele model (G vs. A: *I*^2^ = 32.7%, *P* = 0.216); there was a moderate degree of heterogeneity among studies under the dominant model (GG+AG vs. AA: *I*^2^ = 67.0%, *P* = 0.028) (Figure 4, Supplementary Figure 3).

#### rs12593229

High heterogeneity among studies (Chappell et al., 2011; Zhou et al., 2012; Arja et al., 2014) was detected in the dominant model (TT+GT vs. GG: *I*^2^ = 75.0%, *P* = 0.018), recessive model (TT vs. GG+GT: *I*^2^ = 75.0%, *P* = 0.018), additive model (TT vs. GG; *I*^2^ = 83.7%, *P* = 0.002), and allele model (T vs. G: *I*^2^= 80.5%, *P* = 0.006) (Supplementary Figure 4).

#### rs13180

High heterogeneity among studies (Zhou et al., 2012; Ding et al., 2015; Ziolkowska-Suchanek et al., 2015; Korytina et al., 2016) was detected in the dominant model (TT+ CT vs. CC: *I*^2^ = 62.5%, *P* = 0.046), recessive model (TT vs. CC+CT: *I*^2^ = 76.4%, *P* = 0.005), additive model (TT vs. CC: *I*^2^ = 77.7%, *P* = 0.004); a high degree of heterogeneity was also found among studies (Cho et al., 2012; Hardin et al., 2012; Zhou et al., 2012; Ding et al., 2015; Ziolkowska-Suchanek et al., 2015; Korytina et al., 2016; Nedeljkovic et al., 2018) under the allele model (T vs. C: *I*^2^ = 84.2%, *P* = 0.000) (Supplementary Figure 5).

### Meta-Analysis Results

#### rs2568494

A fixed effects model was used to analyze the dominant model; the recessive, additive, and allele models were analyzed with a random effects model. The results showed a significant difference between COPD patients and controls for the dominant model (AA+GA vs. GG: OR = 1.150, 95% CI: 1.5–1.304, *P* = 0.029). No significant associations were found under the recessive model (AA vs. GG+GA: OR = 0.941, 95% CI: 0.556–1.593; *P* = 0.822), additive model (AA vs. GG; OR = 1.125, 95% CI: 0.0.746–1.696; *P* = 0.575), and allele model (A vs. G; OR = 1.049, 95% CI: 0.880–1.251; *P* = 0.590) between patients and controls (Figure 2, Supplementary Figure 1).

#### rs2656069

To analyze the recessive model using the fixed effects model; the dominant, additive, and allele models using the random effects model. There had a significant difference between COPD patients and controls for the recessive model (GG vs. AA+AG: OR = 0.589, 95% CI: 0.440–0.789; *P* = 0.000), additive model (GG vs. AA: OR = 0.641, 95% CI: 0.441–0.931; *P* = 0.020) and allele model (G vs. A: OR = 0.812, 95% CI: 0.668–0.988; *P* = 0.037). There was no significant associations under the dominant model (GG+AG vs. AA: OR = 0.884, 95% CI: 0.682–1.145, *P* =0.351) between patients and controls (Figure 3, Supplementary Figure 2).

#### rs10851906

The recessive and additive models were analyzed by the fixed effects model; the dominant and allele models were analyzed with a random effects model. The results indicated significant associations between COPD patients and controls for the recessive model (GG vs. AA+AG: OR = 0.732, 95% CI: 0.560–0.958; *P* = 0.023) and additive model (GG vs. AA: OR = 0.777, 95% CI: 0.637–0.947; *P* = 0.012). We did not observe significant associations under the dominant model (GG+AG vs. AA: OR = 0.868, 95% CI: 0.618–1.220, *P* = 0.416) and allele model (G vs. A: OR = 0.903, 95% CI: 0.794–1.026; *P* = 0.118) between patients and controls (Figure 4, Supplementary Figure 3).

#### rs12593229

After meta-analysis with random effects. We did not identify significant difference between COPD patients and controls under the dominant model (TT+GT vs. GG: OR = 0.882, 95% CI: 0.628–1.238, *P* =0.468), recessive model (TT vs. GG+GT: OR = 0.882, 95% CI: 0.605–1.285; *P* = 0.513), additive model (TT vs. GG: OR = 0.854, 95% CI: 0.551–1.326; *P* = 0.483) and allele model (T vs. G: OR = 0.891, 95% CI: 0.699–1.134; *P* = 0.348) between patients and controls (Supplementary Figure 4).

#### rs13180

we performed a meta-analysis using random effects. There were no significant difference between COPD patients and controls under the dominant model (TT + CT vs. CC: OR = 1.106, 95% CI: 0.821–1.492, *P* = 0.507), recessive model (TT vs. CC+ CT: OR = 1.133, 95% CI: 0.816–1.572; *P* = 0.457), additive model (TT vs. CC: OR = 1.170, 95% CI: 0.746–1.836; *P* = 0.495), and allele model (T vs. C: OR = 0.934, 95% CI: 0.767–1.137; *P* = 0.526) between patients and controls (Supplementary Figure 5).

### Publication Bias

**Figure 5:**
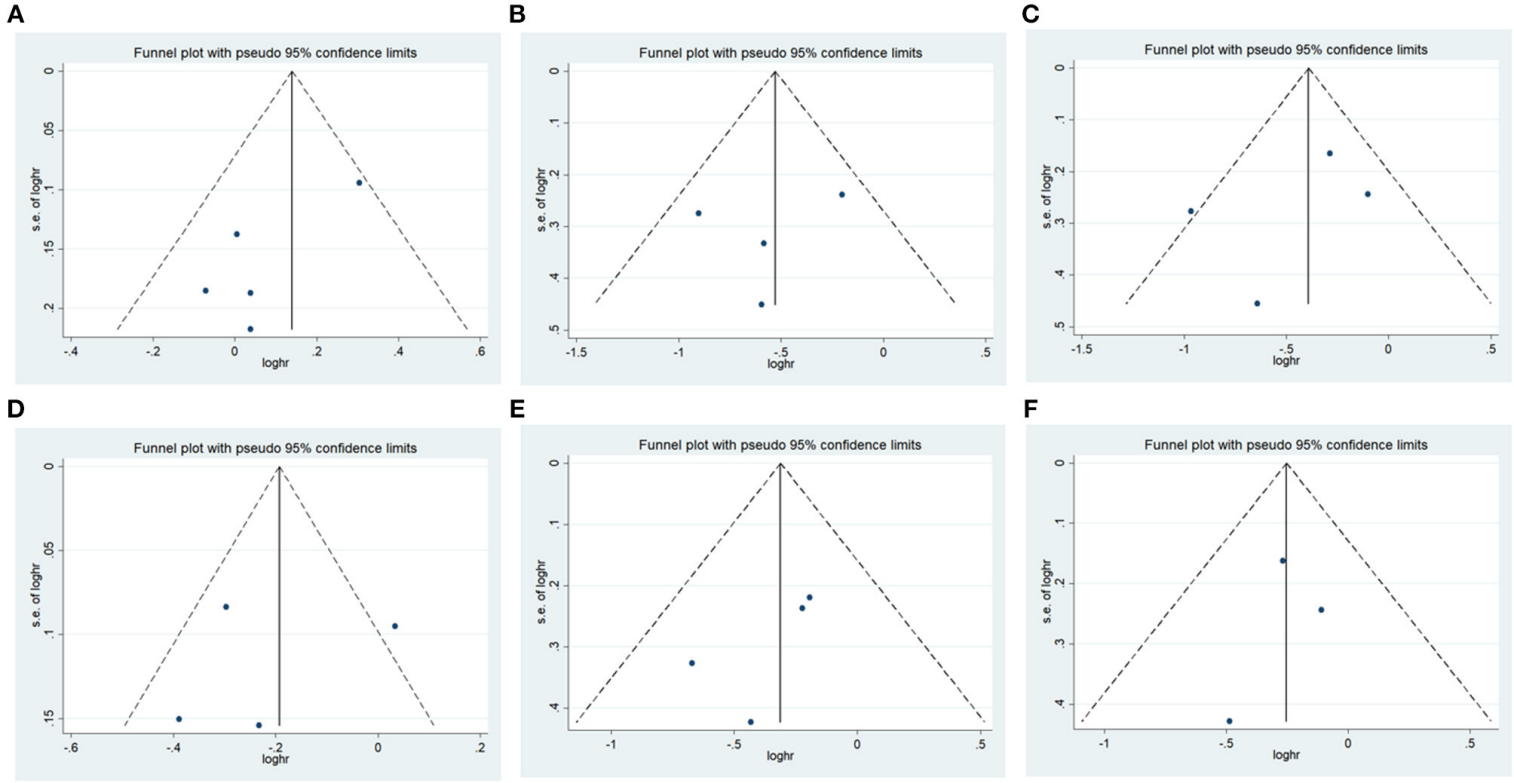
Funnel plot of the odds ratios in the meta-analysis. **(A)** IREB2 rs2568494 dominant model, AA + GA vs. GG. **(B)** IREB2 rs2656069 recessive model, GG vs. AA + AG. **(C)** IREB2 rs2656069 additive model, GG vs. AA. **(D)** IREB2 rs2656069 allele model, G vs. A. **(E)** IREB2 rs10851906 recessive model, GG vs. AA + AG. **(F)** IREB2 rs10851906 additive model, GG vs. AA.

There was no significant publication bias in any of the genetic models according to Begg's and Egger's tests (all *P* > 0.05, data not shown), and the funnel plot was symmetrical, as the studies did not coagulate into one quadrant of the funnel (Figure 5).

## Discussion

Genetic factors play a major role in COPD susceptibility. The region of chromosome 15q25 includes several genes that influence the development of COPD. Among them, *IREB2* is particularly attractive for studying the genetic factors of COPD. In this study, 5 SNPs among COPD patients and healthy controls from previous studies were investigated to evaluate the association of *IREB2* polymorphism with COPD. The results showed that *IREB2* rs2568494 was associated with COPD susceptibility. The minor alleles G by rs2656069 and rs10851906 were protective factors for COPD.

Here, the results showed a significant association of rs2568494 with COPD under the dominant model (AA+GA vs. GG: OR = 1.150, *P* = 0.029). This result has confirmed previous observations suggesting that *IREB2* rs2568494 may play a role in COPD (DeMeo et al., 2009). In addition, two other SNPs (rs2656069 and rs10851906) in *IREB2* have been previously reported, and the minor alleles (G) for both of these SNPs were associated with a decreased risk of COPD. Our results also showed a significant difference between COPD patients and controls for the recessive model (GG vs. AA+AG: OR = 0.589, *P* =0.000) additive model (GG vs. AA: OR = 0.641, *P* = 0.020), and allele model (G vs. A: OR = 0.812, *P* = 0.037) in rs2656069. There was a significant difference between COPD patients and controls for the recessive model (GG vs. AA+AG: OR = 0.732, *P* = 0.023) and additive model (GG vs. AA: OR = 0.777, *P* = 0.012) in rs10851906. These results showed that GG for both SNPs was associated with a decreased risk of COPD, which was in the same direction as previous reports (Qiu et al., 2011). However, we found a lack of association between *IREB2* rs12593229 and rs13180 and COPD susceptibility. Thus, our meta-analysis provides further evidence to support the role of the *IREB2* genomic region in COPD pathogenesis, and the role of *IREB2* deserves further investigation.

There are still some limitations in our study. On the one hand, due to the limited examination of *IREB2* variants in COPD, the sample sizes were comparatively small, which might not provide sufficient statistical power to assess the association between the *IREB2* gene and COPD susceptibility; thus, there is an urgent need to conduct research using large samples across the world. On the other hand, owing to the limitation of the original information, we did not perform a smoking status-stratified analysis, which have the impact on association analysis. We known that the genetic factors of COPD may be complex, with the contribution of environmental factors and multiple genes (Seifart and Plagens, 2007). Thus, it is an important prompt for future study to evaluate whether other risk factors together with the *IREB2* gene influence COPD susceptibility.

## Conclusion

To our knowledge, this is the comprehensive study to assess the role of the *IREB2* rs2568494, rs2656069, rs10851906, rs12593229, and rs13180 variants in COPD. The current results show that there was a significant association between the *IREB2* gene rs2568494 polymorphism and susceptibility to COPD, suggesting that the presence of minor alleles A may be a genetic factor in susceptibility to COPD. Rs2656069 and rs10851906 were associated with a decreased risk of COPD, and the minor alleles G of both SNPs may appear to have a protective effect.

## Author Contributions

QZ, QC, and DZ were responsible for the statistical analysis, study design, and manuscript preparation. RG, DX, and SJ managed the literature searches and analyses. The study was supervised by RC, YW, and GM. All authors contributed to the article and approved the submitted version.

## Conflict of Interest

The authors declare that the research was conducted in the absence of any commercial or financial relationships that could be construed as a potential conflict of interest.
