# Supplementary material for: Different Associations Between the IREB2 Variants and Chronic Obstructive Pulmonary Disease Susceptibility
Source: Front Genet. 2020 Nov 16;11:598053. doi: 10.3389/fgene.2020.598053 (PMC7701307; doi:10.3389/fgene.2020.598053)
Supplement: Supplementary file 1 [file Data_Sheet_1.docx]

**A**

**B**

**C**

FIGURE S1. Meta-analysis with random effects model for the association between the IREB2 rs2568494 polymorphism and COPD susceptibility. (A) Recessive model, AA vs GG+GA; (B) Additive model, AA vs GG; (C) Allele model, A vs G. OR: odds ratio, CI: confidence interval, I-squared: measure to quantify the degree of heterogeneity in meta-analyses.

FIGURE S2. Meta-analysis with random effects model for the association between the IREB2 rs2656069 polymorphism and COPD susceptibility. (Dominant model, GG+AG vs AA). OR: odds ratio, CI: confidence interval, I-squared: measure to quantify the degree of heterogeneity in meta-analyses.

**A**

**B**

FIGURE S3. Meta-analysis for the association between the IREB2 rs10851906 polymorphism and COPD susceptibility. (A) Dominant model, GG+AG vs AA. (random effects model); (B) Allele model, G vs A. (fixed effects model). OR: odds ratio, CI: confidence interval, I-squared: measure to quantify the degree of heterogeneity in meta-analyses.

**A**

**B**

**C**

**D**

FIGURE S4. Meta-analysis with random effects model for the association between the IREB2 rs12593229 polymorphism and COPD susceptibility. (A) Dominant model, TT+GT vs GG; (B) Recessive model, TT vs GG+GT; (C) Additive model, TT vs GG; (D) Allele model, T vs G. OR: odds ratio, CI: confidence interval, I-squared: measure to quantify the degree of heterogeneity in meta-analyses.

**A**

**B**

**C**

**D**

FIGURE S5. Meta-analysis with random effects model for the association between the IREB2 rs13180 polymorphism and COPD susceptibility; (A) Dominant model, TT+CT vs CC. (B) Recessive model, TT vs CC+ CT; (C) Additive model, TT vs CC; (D) Allele model, T vs C. OR: odds ratio, CI: confidence interval, I-squared: measure to quantify the degree of heterogeneity in meta-analyses.
